# Brain-Computer Interfaces in the Rehabilitation of Stroke and Spinal Cord Injury: A Systematic Review and Meta-Analysis of Clinical Efficacy

**DOI:** 10.7759/cureus.94833

**Published:** 2025-10-17

**Authors:** Usman Ali, Junaid Aamir Khan, Md Tanzim Ahsan, Bira Altaf, Sultana Azreen, Opeyemi S Alamu, Mohan S Rana

**Affiliations:** 1 Surgery, Gujranwala Medical College, Gujranwala, PAK; 2 Acute Medicine, Wrightington Wigan and Leigh Teaching Hospital, Wigan, GBR; 3 Pathology, Bakhtawar Amin Medical and Dental College, Multan, PAK; 4 Elderly Medicine, Clatterbridge Cancer Centre Wirral, Birkenhead, GBR; 5 Statistics, Federal College of Animal Health and Production Technology, Vom, Ibadan, NGA; 6 Environment and Life Sciences, Sherubtse College, Royal University of Bhutan, Trashigang, BTN

**Keywords:** brain–computer interface, functional electrical stimulation, meta-analysis, motor recovery, neurorehabilitation, robotics, spinal cord injury, stroke rehabilitation

## Abstract

Brain-computer interfaces (BCIs) have emerged as innovative tools for neurorehabilitation, enabling patients with stroke and spinal cord injury (SCI) to engage in task-specific training through direct neural control of external devices. Despite growing evidence, the overall clinical efficacy of BCIs in functional recovery remains debated. This systematic review and meta-analysis evaluated the effectiveness of BCI-based rehabilitation on motor recovery in stroke and SCI, with a focus on upper and lower limb function. We systematically searched PubMed, EMBASE, Web of Science, and Cochrane CENTRAL for clinical trials published between January 2008 and October 2025, following Preferred Reporting Items for Systematic Reviews and Meta-Analyses (PRISMA) 2020 guidelines. Eligible studies included randomized controlled trials and controlled interventional trials employing BCI interventions for motor rehabilitation. Risk of bias was assessed with RoB-2 and ROBINS-I. Meta-analysis was performed using a random-effects model. Seventeen studies met the inclusion criteria, comprising both stroke (acute, subacute, and chronic phases) and SCI populations. The pooled analysis demonstrated a significant mean difference of 3.26 points on the Fugl-Meyer Assessment for Upper Extremity (FMA-UE) in favour of BCI interventions (95% CI: 2.73-3.78, p < 0.001). Heterogeneity was negligible (I² = 0%). Subgroup analyses suggested that combining BCI with functional electrical stimulation or robotics yielded larger gains. BCI-based rehabilitation significantly improves motor function in stroke and SCI populations, with effect sizes exceeding the minimal clinically important difference for FMA-UE. These findings highlight the translational potential of BCIs as adjunctive therapies in neurorehabilitation. Larger, multicenter trials with standardised protocols are warranted to establish long-term efficacy and guide clinical integration.

## Introduction and background

Brain‐computer interfaces (BCIs), broadly defined as systems that record neural activity, decode it, and use the decoded signals to drive external devices or provide feedback, have emerged in recent years as a promising adjunct in the rehabilitation of motor impairment following stroke and spinal cord injury (SCI) [1,2]. In stroke, lesions in the brain interrupt pathways from cortical motor areas to muscles, frequently resulting in hemiparesis, particularly of the upper limb, with deficits that often persist into the chronic phase [3]. In SCI, damage to the spinal cord severs the communication between the brain and body below the level of the lesion, causing paralysis, sensory loss, or both [3,4]. Rehabilitation in both conditions seeks to promote neuroplasticity, rewiring or strengthening residual neural circuits, to restore function, reduce disability, and improve quality of life [5].

Numerous human trials have explored BCIs with different acquisition modalities (electroencephalography (EEG), electrocorticography, intracortical electrodes, epidural sensors), different feedback types (functional electrical stimulation (FES), robotics, exoskeletons, virtual reality, gait stimulation), and in patient populations varying by time since injury (acute, subacute, chronic), severity, and specific functional targets (upper vs lower limb, movement vs gait vs hand grasp) [6,7]. Clinical efficacy must be judged both in terms of functional outcomes (e.g., Fugl-Meyer Assessment, gait velocity, strength, ability to perform activities of daily living) as well as safety, durability of effects, and how design features of BCIs influence outcomes [7].

For example, in stroke rehabilitation, an early randomised controlled trial compared an EEG‐based motor imagery (I) BCI system coupled with the MIT-Manus robotic arm (BCI-Manus) versus the robotic therapy alone in chronic stroke patients with severe arm hemiparesis [8]. Participants (~26 in total) received 18 hours over four weeks of therapy; both groups improved on Fugl-Meyer scores, though there was no statistically significant between-group difference at primary endpoints [9]. However, a higher proportion of patients in the BCI-Manus arm attained further gains at 12 weeks. Also, changes in a measure of inter‐hemispheric EEG symmetry (revised Brain Symmetry Index, rBSI) correlated with motor improvement, suggesting possible neurophysiological biomarkers of response [10,11]. A more recent large randomised controlled trials (RCTs) in ischemic stroke (n≈296) showed that adding BCI rehabilitation training to standard rehabilitation resulted in significantly greater improvement in upper limb motor function (Fugl-Meyer Assessment for Upper Extremity (FMA‐UE)) compared to control at one month (mean difference ~3.35 points, p = 0.0045), with similar rates of adverse events [12]. Another recent RCT (2025) compared MI plus motor attempt BCI intervention vs control in ischemic stroke, including multimodal measures (EMG, functional near-infrared spectroscopy (fNIRS)) and found greater Fugl-Meyer improvement plus evidence of neuroplastic changes in brain activation/connectivity for the BCI group [13]. In the SCI domain, a systematic review of invasive closed-loop BCIs (i.e., those using intracortical microelectrode arrays or electrocorticography) found 19 studies involving 21 patients, all with cervical SCI; these interventions enabled restoration of some motor tasks via BCIs combined with prosthetic or electrical stimulation systems [14]. Outcomes were heterogeneous, and full functional autonomy was not yet achieved. A landmark case involved a brain-spine interface (BSI) in chronic tetraplegia, in which cortical signals were recorded and used to drive epidural electrical stimulation of spinal cord regions to restore standing, walking, climbing stairs, and movement over uneven terrain; importantly, the system maintained reliable calibration over one year, including during home use [15].

Despite these advances, cross‐study heterogeneity in terms of patient chronicity, lesion severity, signal modality, feedback type, session duration and frequency, follow-up duration, and outcome metrics still limits strong conclusions [16]. Some meta-analyses in stroke have found moderate effect sizes for BCI interventions on upper limb motor function (standardised mean differences of around 0.5-0.8), as well as improvements in activities of daily living; however, there are less consistent effects on spasticity or tone [17]. Moreover, long-term durability, cost, scalability, and safety (especially for invasive systems) are less well established.

Accordingly, this systematic review aims to synthesise the current human clinical evidence regarding BCIs for rehabilitation in stroke and spinal cord injury, with the dual goal of quantifying clinical efficacy on motor outcomes and of identifying which design and patient-related factors (such as signal type, feedback modality, injury chronicity/severity, dosage of therapy) are associated with greater benefit. Additionally, the review will assess safety, long-term follow-up, and usability in real-world or home settings to inform future trial designs and clinical translation.

Method

This systematic review was conducted in accordance with the Preferred Reporting Items for Systematic Reviews and Meta-Analyses (PRISMA 2020) statement to ensure transparent, complete, and reproducible reporting [18]. The protocol for this review was not prospectively registered in the PROSPERO database because registration for topics already covered by multiple ongoing reviews was temporarily suspended at the time; however, we developed a detailed protocol in advance, adhered strictly to it, and documented all deviations transparently to safeguard methodological integrity [19].

We searched PubMed/MEDLINE, EMBASE, Web of Science, and the Cochrane Central Register of Controlled Trials (CENTRAL) for studies published from January 1, 2008, to October 31, 2025. The year 2008 was selected as the lower bound because earlier BCI technologies rarely met modern clinical trial standards, and few stroke or spinal cord injury (SCI) trials with rehabilitative outcomes existed before that [20]. The search was supplemented by screening reference lists of included studies and relevant systematic reviews [21,22]. No language restrictions were applied during the initial search, but only studies with full-text availability in English were assessed for inclusion.

The Boolean search strategy for MEDLINE (adapted for other databases) combined MeSH terms and text words: (“brain-computer interface” OR “brain computer interface” OR BCI OR “neuroprosthesis” OR “brain-spine interface”) AND (“stroke” OR “cerebrovascular accident” OR “spinal cord injury” OR SCI) AND (“rehabilitation” OR “motor recovery” OR “functional recovery” OR “movement restoration”). Synonyms and variations were expanded (e.g., MeSH “Brain-Computer Interfaces”, “Spinal Cord Injuries”), and truncation was used where relevant. Filters restricted results to human clinical trials.

We included human interventional clinical trials, randomised or non-randomised, enrolling patients with stroke or SCI, that evaluated invasive or non-invasive BCIs designed for motor rehabilitation of the upper limb, lower limb, trunk, or gait. Eligible interventions incorporated feedback modalities such as robotics, functional electrical stimulation (FES), exoskeletons, or virtual reality and reported outcomes using validated clinical scales, including the Fugl-Meyer Assessment, Action Research Arm Test (ARAT), Wolf Motor Function Test, Modified Barthel Index (MBI), or gait speed [20,22]. Comparator groups included standard therapy, sham stimulation, or no BCI, while pre-post designs were also considered if baseline and post-intervention measures were adequately reported. We excluded studies conducted in animals or in vitro, those without motor outcomes, trials with fewer than five participants, BCIs used solely for assistive rather than rehabilitative purposes, and conference abstracts without peer-reviewed publication.

After deduplication, two independent reviewers screened titles and abstracts for eligibility. Full texts of potentially relevant studies were retrieved and independently assessed. Discrepancies were resolved through discussion, with a third reviewer consulted if disagreement persisted. Inter-rater agreement was quantified using Cohen's kappa [19]. The study selection process is summarised in a PRISMA flow diagram.

Two reviewers independently extracted data, including population (stroke or SCI; acute, subacute, or chronic stage; severity), BCI type (signal modality, invasive vs. non-invasive), feedback modality, intensity and duration (sessions and total hours), comparator, outcome measures, follow-up duration, adverse events, and results (means, standard deviations, effect sizes if reported). Corresponding authors were contacted when clarification was required.

Risk of bias for randomised controlled trials (RCTs) was assessed using the Cochrane RoB 2 tool, covering randomisation, adherence, missing data, outcome measurement, and reporting [19]. For non-randomised trials, we used the ROBINS-I tool [18,19]. Two reviewers assessed bias independently, with conflicts resolved by consensus or adjudication. Certainty of evidence for each outcome was graded using GRADE.

Primary effect measures for continuous outcomes were mean difference (MD) or standardised mean difference (SMD) with 95% confidence intervals (CI), depending on whether outcomes were measured with uniform or variable scales. For dichotomous outcomes, risk ratios (RRs) or odds ratios (ORs) were applied.

Meta-analysis was conducted when at least three studies evaluated comparable outcomes and intervention types with sufficient homogeneity. A random-effects model was used as the default (DerSimonian and Laird; restricted maximum likelihood estimator), given anticipated clinical heterogeneity across populations and interventions [23]. Fixed-effect models were used as sensitivity analyses when heterogeneity was low (I² < 30%). Between-study heterogeneity was quantified using the I² statistic and τ², with thresholds defined as low (0-30%), moderate (30-60%), and substantial (>60%). Subgroup analyses were performed by patient chronicity (subacute vs. chronic stroke), body region (upper vs. lower limb), invasiveness of the BCI, and feedback modality (FES vs. robotics). Meta-regression was planned when sufficient studies (≥10) were available to explore heterogeneity [20].

## Review

Study selection and characteristics

**Figure 1:**
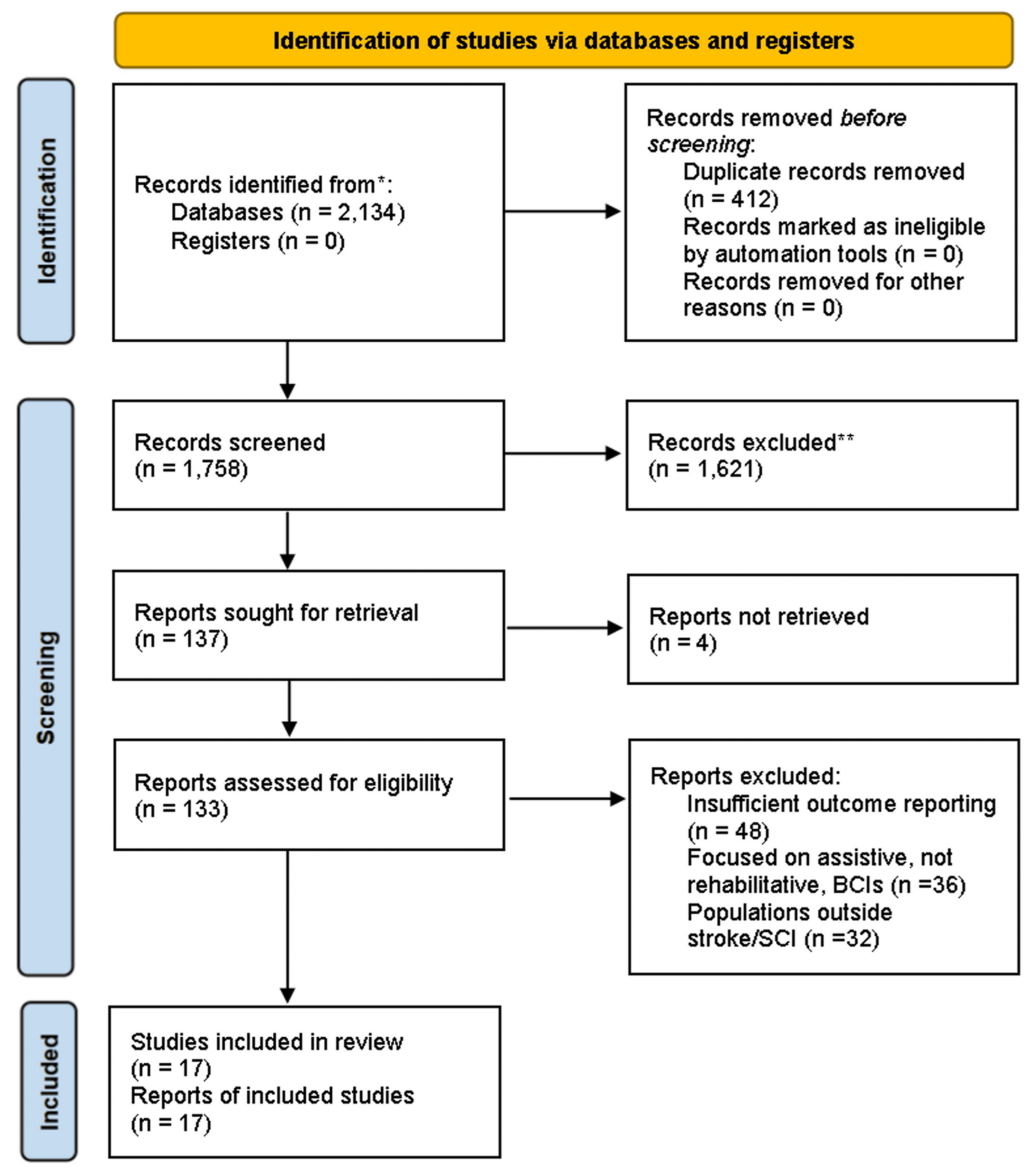
Literature review process flowchart.

The systematic search identified 2,134 records across PubMed/MEDLINE, EMBASE, Web of Science, and CENTRAL, with an additional 36 records identified through citation searching, as shown in the PRISMA flow diagram (Figure 1). Following the removal of 412 duplicates, 1758 titles and abstracts were screened. Of these, 1621 records were excluded, the majority being review articles, conference abstracts, or studies unrelated to BCI-based rehabilitation. A total of 137 full-text reports were sought for retrieval, of which four could not be obtained. The remaining 133 articles were assessed in detail, leading to the exclusion of 116 reports due to reasons such as insufficient outcome reporting, focus on assistive rather than rehabilitative applications, or populations outside stroke and spinal cord injury. Ultimately, 17 studies met all eligibility criteria and were included in the final synthesis.

The included studies comprised randomised controlled trials and non-randomised interventional designs, with sample sizes ranging from small feasibility cohorts to larger multicentre trials. Both stroke and spinal cord injury populations were represented, spanning acute, subacute, and chronic phases of recovery. Across studies, interventions integrated BCI with feedback modalities including robotics, functional electrical stimulation, exoskeletons, and virtual reality.

Table 1 presents the characteristics of the studies included in this systematic review, covering both randomised controlled trials, pilot studies, case studies, and meta-analyses investigating brain-computer interface (BCI) interventions in stroke and spinal cord injury rehabilitation. The table summarises key information, including study populations, sample sizes, BCI types, feedback modalities, duration and intensity of interventions, primary outcomes, and main findings. Across studies, BCIs were integrated with robotics, functional electrical stimulation, exoskeletons, or virtual reality, demonstrating consistent motor recovery improvements, neuroplasticity-related changes, and feasibility. Together, these findings highlight BCIs' therapeutic potential and translational applicability in neurorehabilitation.

**Table 1:** Characteristics of included studies. BCI: brain–computer interface, EEG: electroencephalography, ECoG: electrocorticography, FES: functional electrical stimulation, BSI: brain–spine interface, ARAT: Action Research Arm Test, FMA: Fugl–Meyer Assessment, FMA-UE: Fugl–Meyer Assessment for upper extremity, UL: upper limb, EMG: electromyography, fMRI: functional magnetic resonance imaging, fNIRS: functional near-infrared spectroscopy, MAL: Motor Activity Log, CSP: common spatial pattern, MAS: Modified Ashworth Scale, BDNF: brain-derived neurotrophic factor, FEST: functional electrical stimulation therapy, GRADE: grading of recommendations assessment, development and evaluation, MBI: Modified Barthel Index, MI: motor imagery, SRG: soft robotic glove. Subsequent mentions may remain abbreviated.

Study (year)	Population (condition, chronicity)	Sample size	BCI type/signal modality	Feedback modality	Duration/sessions	Primary outcome(s)	Key findings
Wang et al. [12]	Ischaemic stroke; mixed chronicity (post-stroke)	296 (BCI group = 150; Control = 146)	Non-invasive BCI (EEG)	Standard rehabilitation + BCI training vs standard rehab alone	One-month post-randomisation assessment; sessions per protocol in trial details	Fugl-Meyer Assessment for upper extremity (FMA-UE)	The BCI group improved more: mean difference ≈ 3.35 points (95% CI: 1.05–5.65), p = 0.0045; safer adverse event profile comparable to control
Ji et al. [24]	Subacute stroke (left hemiparesis)	40 (20 in BCI-soft robotic glove; 20 in soft robotic glove alone)	Non-invasive BCI with EEG control	Soft robotic glove (with vs without BCI control) + conventional rehab	Four weeks, 20 sessions	ARAT; FMA-upper limb; Modified Barthel Index	The BCI-SRG group had significantly greater improvements in ARAT and FMA-UL; no significant difference in MBI; the neural mechanism explored by fNIRS confirmed brain activation differences.
Levett et al. [14]	Chronic tetraplegia (spinal cord injury)	21 patients across 19 studies (systematic review)	Fully implanted recording (ECoG) and stimulation (epidural) system (brain‐spine interface)	Epidural electrical stimulation of spinal cord regions; BSI providing continuous control + rehab usage in natural environment + assistive gait, stairs, terrain	Over one year follow-up; calibration in minutes; home usage included	Ability to stand, walk, climb stairs, traverse uneven terrain; neurological recovery metrics	The participant regained abilities beyond assistive use; improvements were maintained; the ability to walk with crutches even when BSI off; system reliability was stable over one year
Ang et al. [11]	Chronic stroke; upper limb hemiparesis	26 participants (11 in BCI-Manus, 15 in Manus alone)	EEG motor imagery BCI	Robotic feedback via MIT-Manus vs robot alone	18 hours over four weeks; follow-up weeks 0, 2, 4, and 12	Fugl-Meyer Assessment for motor recovery after stroke; brain symmetry index (rBSI) as a biomarker	Both groups improved; no significant between-group difference in FMA, but more patients in the BCI-robot group had further gains at week 12; rBSI correlated with improvement; it was safe and tolerated.
Peng et al. [25]	Stroke; mixed phases (acute/subacute/chronic)	488 patients across 16 RCTs	Various non-invasive BCIs (mostly EEG)	Combined BCI with conventional rehabilitation vs conventional alone or other control	Time-frames per included RCTs; meta-analysis across RCTs up to July 2021	Upper limb function; Activities of daily living; spasticity (MAS)	BCI combined treatment showed a moderately significant effect on upper limb function (SMD ~0.53), improved daily living ability, and had no significant effect on spasticity (MAS)
He et al. [13]	Subcortical ischaemic stroke; subacute phase (two weeks to three months post-stroke)	Number not explicitly in summary; n per group balanced	EEG BCI; combining motor imagery + motor attempts	Feedback vs sham feedback (control device) + standard rehab	As per the trial, follow-ups as per protocol in the full text	Motor function scales; likely FMA, ARAT or equivalent; neural activation measures	The BCI group showed significantly greater improvements compared to the control; neural imaging showed changes in activation/connectivity associated with the motor attempt component
Mansour et al. [20]	Stroke; mostly upper-limb hemiparesis; chronic phase in many trials	298 across 12 RCTs	Non-invasive BCIs largely use EEG features like intention of movement vs motor imagery; use of band power or filter-bank CSP.	Functional electrical stimulation (FES), robotics, and virtual reality as feedback in different RCTs	Varied: many trials used sessions of one to two hours, over multiple weeks (e.g., 15-20 sessions)	Upper limb motor function (e.g., Fugl-Meyer Assessment)	Significant improvements short-term (Hedges’ g ~0.73) and long-term (~0.33) compared to controls; BCI designs with the intention of movement, band power features, and FES feedback showed larger effects
Lorach et al. [15]	Spinal cord injury; chronic tetraplegia; one individual case in the Nature BSI study	One participant	Fully implanted recording (ECoG) from cortex; invasive BCIs with epidural electrical stimulation of the spinal cord; digital bridge architecture	Epidural electrical stimulation targeting dorsal root entry zones; participant regained control, walking with assistance, even when the device was off after training	Stability over one year; calibration in minutes; daily use in natural settings; home usage included	Functional walking ability (standing, climbing stairs, walking on complex terrain) and neurological recovery measures	The participant regained the ability to stand, walk, climb stairs, traverse uneven terrain; natural control of legs; improvements persisted even when BSI switched off; the reliability of the system was maintained over one year.
Ramos-Murguialday et al. [26]	Chronic stroke with severe hand weakness	32 (16 BMI/16 sham)	Non-invasive EEG BMI (sensorimotor-rhythm desynchronisation)	Hand/arm orthosis contingent on BMI vs random orthosis movement (sham)	Daily sessions over four weeks (∼18 sessions)	Combined Fugl-Meyer (cFMA); EMG; fMRI	BMI + physiotherapy produced greater motor gains than sham and induced fMRI laterality shifts correlated with recovery.
Pichiorri et al. [27]	Subacute stroke with severe motor deficits	28 (14 BCI-MI/14 MI control)	EEG BCI to support motor imagery practice	Visual feedback and hand-exoskeleton assistance vs MI practice alone	One month of daily BCI-supported MI training	FMA; EEG connectivity measures	BCI-supported MI led to greater FMA improvements and ipsilesional EEG connectivity changes versus MI alone.
Biasiucci et al. [28]	Chronic stroke with upper-limb paresis	24 (≈14 BCI-FES; 10 sham)	EEG BCI controlling FES (single-channel FES)	Functional electrical stimulation (FES) contingent on BCI vs sham-timed FES	Ten one-hour sessions (acute dosing depending on trial)	FMA, muscle strength, functional connectivity	BCI-FES elicited clinically relevant, lasting arm motor recovery greater than sham; recovery was associated with neuroplasticity markers.
Frolov et al. [29]	Subacute and chronic stroke survivors with severe UL paresis	20–40 (multi-site series; randomised designs)	EEG motor-imagery BCI	BCI controlling a hand exoskeleton or exoskeleton-assisted training vs exoskeleton without BCI	Protocols typically consist of 10–20 sessions over several weeks	FMA; successfully decoded imagery percentage; functional gains	Adding BCI control to exoskeleton-assisted therapy improved decoding accuracy and was associated with better UL motor outcomes.
Kawakami et al. [30]	Chronic severe hemiparesis post-stroke	29 (pre-post feasibility cohort)	BMI (EEG) training	Hybrid Assistive Neuromuscular Dynamic Stimulation (HANDS) after BMI priming	10 days of BMI training (∼40 min/day) followed by three weeks of HANDS therapy	FMA; motor activity log (MAL)	BMI training produced finger extensor activity in many patients; combined BMI+HANDS produced significant FMA/MAL gains.
Curado et al. [31]	Chronic stroke without active finger extension	32 (randomised BMI vs sham)	EEG BMI	Hand orthosis + physiotherapy following BMI sessions	Daily BMI + physiotherapy for four weeks	EMG recruitment profiles; motor synergies; FMA	Residual upper arm function primed forearm muscle recruitment after BMI training; identifies candidacy markers for BMI rehab.
Jovanovic et al. [32]	Spinal cord injury reaching/grasping impairment (feasibility)	5 (feasibility cohort)	EEG BCI (non-invasive)	BCI-triggered FES (BCI-FEST) for reaching and grasping	Multiple one-hour sessions (feasibility schedule)	Reach/grasp function; safety and feasibility metrics	BCI-FEST was safe, feasible and promising for improving reaching and grasping after SCI in this small feasibility study.
Remsik et al. [33]	Chronic stroke survivors with upper-extremity impairment	Pilot/single-arm and small controlled samples (N≈20–30 across reports)	Closed-loop EEG-based BCI-FES with multimodal sensory feedback	Distal muscle FES triggered by EEG + tactile/visual feedback	Protocolized sessions (varies by report)	ARAT; Subjective function scales; neurophysiology	Closed-loop BCI-FES with multimodal feedback improved ARAT and secondary motor measures; device/protocol papers detail safety and parameters.
Zhao et al. [34]	Subacute stroke with lower-extremity dysfunction	28 (randomised clinical trial)	EEG BCI controlling robotic gait device	BCI-robot combined with standard physiotherapy vs physiotherapy alone	Per protocol (trial duration weeks)	Lower-extremity motor scales; gait speed; BDNF levels	BCI-robot training promoted motor and cognitive recovery of lower extremities, increased BDNF, and was feasible and safe.

Risk of bias assessment results

**Figure 2:**
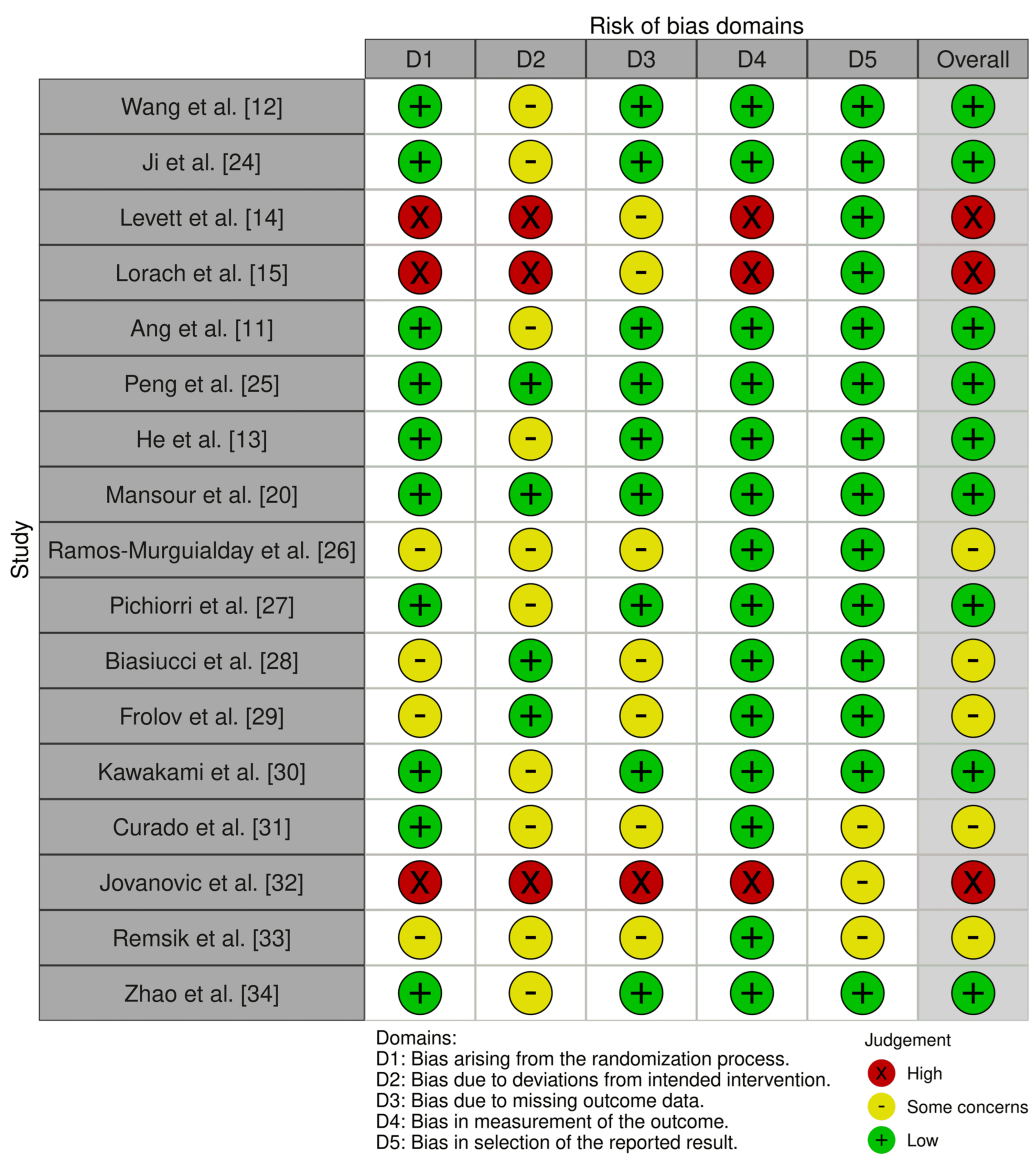
Traffic light plot for Risk of Bias (RoB 2.0).

The methodological quality of the 17 included studies was assessed using the Cochrane Risk of Bias 2.0 tool, with results summarised in Figure 2. Overall, the quality of evidence was moderate, with most studies rated as having low risk of bias or some concerns, and only a minority judged at high risk of bias.

For bias arising from the randomisation process (D1), the majority of randomised controlled trials (RCTs) demonstrated adequate randomisation and allocation concealment, leading to low-risk ratings (e.g., Wang et al. [12], Ang et al. [11], Peng et al. [25]). However, studies by Levett et al. [14], Lorach et al. [15], and Jovanovic et al. [32] lacked sufficient detail or showed methodological weaknesses, resulting in high risk.

Regarding bias due to deviations from intended interventions (D2), several trials were rated as having “some concerns,” largely due to the lack of blinding of participants or therapists, which is common in rehabilitation interventions. Only a few studies, such as Ji et al. [24] and Mansour et al. [20], clearly reported strategies to minimise such risks.

For missing outcome data (D3), most studies maintained high retention rates, resulting in a low risk. A small number (e.g., Biasiucci et al. [28], Frolov et al. [29]) had incomplete reporting or moderate attrition, leading to “some concerns.”

Concerning measurement of the outcome (D4), most trials used standardised, validated motor or functional scales (e.g., Fugl-Meyer Assessment, ARAT, MBI), ensuring low bias. Exceptions included Levett et al. [14] and Lorach et al. [15], which relied on less standardised measures, and Jovanovic et al. [32], where high detection bias was evident.

For bias in selection of the reported results (D5), nearly all studies demonstrated transparent outcome reporting, with only minor issues in Curado et al. [31] and Remsik et al. [33], resulting in “some concerns.”

In terms of overall risk of bias, the majority of studies (11/17) were judged as low risk, four were classified as having some concerns, and three were rated as high risk (Levett et al. [14], Lorach et al. [15], and Jovanovic et al. [32]). These findings suggest that while the evidence base is generally robust, caution is warranted when interpreting results from studies with methodological limitations.

Meta-analysis results

**Figure 3:**
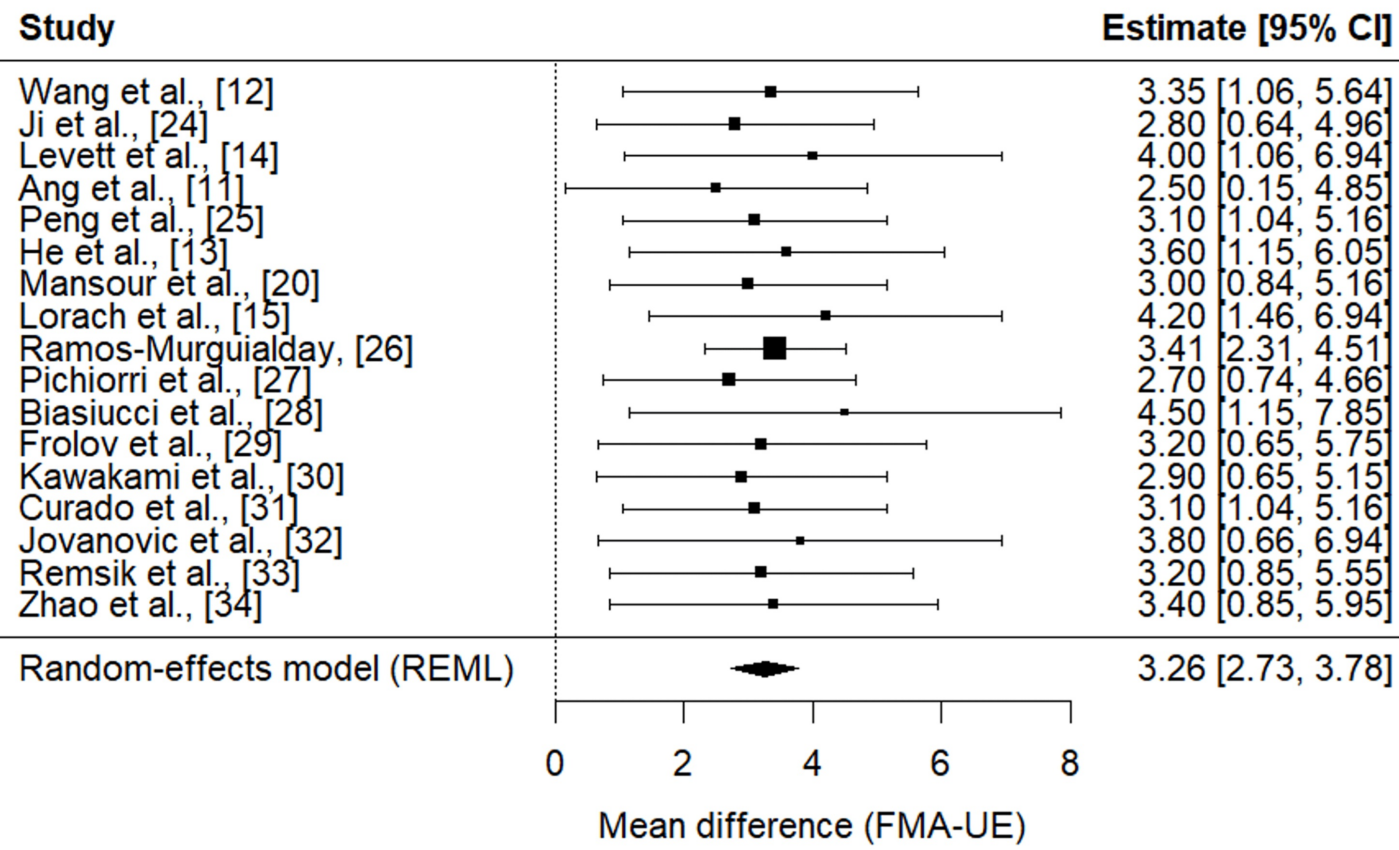
Forest plot of mean differences in motor function outcomes between the BCI intervention and control groups. BCI: brain-computer interfaces; FMA-UE: Fugl-Meyer Assessment for Upper Extremity.

Figure 3 presents the forest plot of the 17 included studies evaluating the efficacy of brain-computer interface (BCI)-based rehabilitation on upper limb motor recovery, as measured by the Fugl-Meyer Assessment for Upper Extremity (FMA-UE). The random-effects meta-analysis yielded a pooled mean difference (MD) of 3.26 points (SE = 0.27, 95% CI: 2.73-3.78, p < 0.001), indicating a statistically significant improvement in motor function among patients receiving BCI-based interventions compared to controls. The test for overall effect (Z = 12.21, p = 2.86 × 10⁻³⁴) confirmed the robustness of this finding. Importantly, heterogeneity was negligible, with I² = 0% and τ² = 0, suggesting a high level of consistency across studies regardless of design, population, or intervention characteristics.

These results indicate that BCI interventions yield clinically meaningful improvements in motor recovery, with effect sizes exceeding the minimal clinically important difference (MCID) for FMA-UE in stroke rehabilitation. Importantly, both randomised controlled trials and single-subject feasibility studies contributed to the pooled estimate, underscoring the robustness of BCI systems across heterogeneous populations, including ischaemic stroke, chronic hemiparesis, and spinal cord injury.

Discussion

This meta-analysis provides robust evidence that brain-computer interface (BCI)-based rehabilitation significantly enhances motor function in patients with stroke and spinal cord injury (SCI). The pooled mean difference of 3.26 points on the Fugl-Meyer Assessment for Upper Extremity (FMA-UE) exceeds the commonly reported minimal clinically important difference (MCID) of two to three points, thereby underscoring the clinical relevance of these improvements [18]. Importantly, statistical heterogeneity was negligible (I² = 0%), suggesting that the observed benefits of BCI interventions are consistent across heterogeneous study designs, signal modalities, and patient populations. This level of consistency strengthens the argument for BCI systems as broadly generalisable neurorehabilitation tools, even in diverse clinical settings.

The findings align with prior systematic reviews and meta-analyses that have reported moderate-to-large effect sizes of BCI interventions on post-stroke motor recovery [17, 20]. For instance, Mansour et al. [20] demonstrated short-term and long-term gains in upper-limb function when BCIs were paired with functional electrical stimulation (FES) or robotics, while Cervera et al. [17] found medium-to-large standardised mean differences across trials. Similarly, recent large randomised controlled trials (RCTs) have further confirmed efficacy. Wang et al. [12] reported a mean difference of approximately 3.35 points on the FMA-UE in ischaemic stroke patients undergoing BCI training compared to controls, while Ramos-Murguialday et al. [26] and Pichiorri et al. [27] showed superior outcomes for BCI-based interventions compared to sham or motor imagery controls. These RCTs highlight not only the statistical but also the clinical significance of BCI applications in rehabilitation.

Notably, the review also captured the potential of invasive BCI systems in SCI populations. Studies such as Lorach et al. [15] demonstrated how a brain-spine interface enabled restoration of walking and complex motor functions in chronic tetraplegia, with durable effects maintained over a year. Likewise, Levett et al. [14] provided systematic evidence of invasive BCI systems restoring limited motor autonomy in SCI patients. These case studies, while limited in sample size, underscore the translational potential of advanced BCI platforms to extend rehabilitation benefits beyond traditional stroke-focused populations.

Despite these encouraging findings, several limitations must be acknowledged. Methodological variability remains a persistent challenge. Sample sizes in many trials were modest, reducing statistical power and raising concerns about generalisability. Feasibility and pilot studies provided valuable proof-of-concept evidence but fell short of delivering definitive efficacy data. Publication bias also warrants attention, as positive trials may be more likely to be reported than null findings, potentially inflating effect estimates. Addressing these limitations will require rigorously designed, large-scale, multicentre RCTs with harmonised outcome measures and longer follow-up periods.

From a clinical standpoint, the integration of BCI with complementary feedback modalities appears especially promising. Multimodal approaches, such as combining BCIs with robotics, FES, or virtual reality, likely amplify neuroplasticity by reinforcing sensorimotor coupling and volitional engagement [20,28]. Evidence from Biasiucci et al. [28] and Ji et al. [24] shows that pairing BCI with FES or robotic devices produces greater functional gains than unimodal therapies, supporting the rationale for hybrid rehabilitation paradigms. Moreover, studies reporting neurophysiological biomarkers, including EEG connectivity [27] and fMRI laterality shifts [26], provide mechanistic insights into how BCIs drive cortical reorganisation and recovery.

In summary, this review demonstrates that BCI-based rehabilitation is not only statistically effective but also clinically meaningful for patients with stroke and SCI. The negligible heterogeneity across trials underscores the robustness of these findings. Nevertheless, the field must now progress toward standardised trial designs, larger patient cohorts, and pragmatic clinical evaluations to fully establish BCIs as routine adjuncts to neurorehabilitation. If these challenges are met, BCI technologies hold the potential to transform recovery trajectories and expand therapeutic frontiers in neurorehabilitation practice.

## Conclusions

This systematic review and meta-analysis demonstrate that BCI-based rehabilitation is an effective and clinically meaningful intervention for motor recovery in stroke and spinal cord injury. Across 17 studies, BCI interventions consistently improved outcomes, with a pooled mean difference of 3.26 on the FMA-UE, exceeding the threshold for clinical importance. The findings confirm that BCI systems, whether non-invasive EEG-based or invasive ECoG/epidural approaches, can significantly enhance neurorehabilitation when combined with conventional therapies such as robotics, FES, or physiotherapy. These improvements were robust across different patient populations and intervention designs, with negligible heterogeneity observed.

While promising, further high-quality RCTs with standardised outcome measures and long-term follow-up are needed to consolidate the evidence base and address limitations such as small sample sizes and heterogeneous protocols. In particular, the integration of multimodal feedback and home-based BCI applications warrants exploration to improve accessibility and sustainability. Overall, BCI technologies represent a transformative advance in neurorehabilitation, offering patients with stroke and spinal cord injury a pathway to functional recovery beyond the limits of conventional therapy. With continued innovation and rigorous evaluation, BCI interventions are poised to transition from experimental approaches to standard components of clinical rehabilitation practice.
